# Assessment of Prescription Opioid Medical Use and Misuse Among Parents and Their Adolescent Offspring in the US

**DOI:** 10.1001/jamanetworkopen.2020.31073

**Published:** 2021-01-07

**Authors:** Pamela C. Griesler, Mei-Chen Hu, Melanie M. Wall, Denise B. Kandel

**Affiliations:** 1 Department of Psychiatry, Columbia University Vagelos College of Physicians and Surgeons, New York, New York; 2 New York State Psychiatric Institute, New York, New York; 3 Department of Biostatistics, Columbia University Mailman School of Public Health, New York, New York; 4 Research Foundation for Mental Hygiene, New York, New York; 5 Department of Sociomedical Sciences, Columbia University Mailman School of Public Health, New York, New York

## Abstract

**Importance:**

Limited information is available regarding the association between parental and adolescent medical prescription opioid use and misuse in the US.

**Objective:**

To examine the associations between parental and adolescent prescription opioid medical use and misuse.

**Design, Setting, and Participants:**

This cross-sectional, nationally representative study included 15 200 parent-adolescent dyads from the annual 2015-2017 National Survey on Drug Use and Health. Data were collected from January 6, 2015, to December 20, 2017, and analyzed from October 4, 2019, to October 15, 2020.

**Exposures:**

Parental past 12-month exclusive medical prescription opioid use and any misuse (ie, using without a prescription or in any way not directed by a physician).

**Main Outcomes and Measures:**

Adolescent past 12-month medical prescription opioid use or misuse. Multivariable regressions estimated associations between parental and offspring medical prescription opioid use or misuse, controlling for sociodemographic and psychosocial variables.

**Results:**

Respondents included 9400 mother-child and 5800 father-child dyads in the same household; children were aged 12 to 17 years (52.8% male; mean [SD] age, 14.5 [1.7] years). Controlling for other factors, parental medical prescription opioid use was associated with adolescent prescription opioid medical use (adjusted odds ratio [aOR], 1.28; 95% CI, 1.06-1.53) and misuse (aOR, 1.53; 95% CI, 1.07-2.25), whereas parental misuse was not. Parental medical prescription stimulant use was associated with adolescent medical prescription opioid use (aOR, 1.40; 95% CI, 1.02-1.91). Parental marijuana use (aOR, 1.84; 95% CI, 1.13-2.99), parent-adolescent conflict (aOR, 1.26; 95% CI, 1.05-1.52), and adolescent depression (aOR, 1.75; 95% CI, 1.26-2.44) were associated with adolescent prescription opioid misuse. Adolescent delinquency (aOR, 1.55; 95% CI, 1.38-1.74) and perceived schoolmates’ drug use (aOR, 2.87; 95% CI, 1.95-4.23) were also associated with adolescent misuse and more weakly with medical use (aORs, 1.13 [95% CI, 1.05-1.22] and 1.61 [95% CI, 1.32-1.96], respectively).

**Conclusions and Relevance:**

Youth use of prescription opioids is in part a structural/environmental issue. The findings of this study suggest that parental medical prescription opioid use is associated with offspring prescription opioid use, whereas parental misuse is not. Restricting physicians’ opioid prescribing to parents is a crucial public health goal. In addition, parents could be educated on the risks of their prescription opioid use for offspring and on practices to mitigate risk, including safe medication storage and disposal. Screening for parental prescription opioid use could be part of pediatric practice. Addressing adolescent mental health could also reduce adolescent prescription opioid misuse.

## Key Points

**Question:**

What are the patterns of association between parental and adolescent medical prescription opioid use and misuse in the US?

**Findings:**

In this nationally representative cross-sectional study of 15 200 parent-adolescent dyads from the National Survey on Drug Use and Health, controlling for other factors, parental medical prescription opioid use within the past year was associated with adolescent medical prescription opioid use and misuse, whereas parental misuse was not.

**Meaning:**

These findings suggest that reducing opioid prescribing by physicians and educating families about medication use practices that restrict the availability of prescription opioids to adolescents in the home could be important targets for reducing adolescent prescription opioid misuse.

## Teaser

This cross-sectional study examines the associations between parental and adolescent prescription opioid medical use and misuse among parent and adolescent dyads in the National Survey on Drug Use and Health.

## Introduction

Parental smoking and alcohol and marijuana use are associated with increased use of the same drug by offspring,^1,2,3,4^ as is prescription opioid misuse.^5^ However, limited information is known to date about the specific associations between parental and adolescent self-reported medical prescription opioid use, in addition to misuse, especially at the national level. We previously examined parent-adolescent prescription opioid misuse from 2004 to 2012 in the National Survey on Drug Use and Health (NSDUH), when medical use was not assessed.^5^ Existing studies have examined the associations between opioids prescribed to family members and other household members in administrative commercial insurance claims. Exposure to family members’ opioid prescriptions is associated with increased initiation of prescription opioid use among family members,^6^ persistent opioid use after surgery among youths,^7^ and opioid overdoses among youths who had not been prescribed opioids, especially at high dosages.^6,7,8,9,10^ The family member’s relationship to the study populations is not specified except for mothers in one study^9^ and parents in another.^7^ These studies do not inform on the specific role of parental prescription opioid medical use and misuse in adolescent misuse or on parental and adolescent characteristics that could also account for youth misuse. They highlight, however, the crucial role of opioid prescribing to family members in opioid use or overdose by another family member. As emphasized by Seamans et al,^6^^(p103)^ “Opioids are often prescribed in doses exceeding clinical guidelines for patients with non-cancer related pain, and in large quantities, resulting in surpluses of opioids stored in household medicine cabinets. Unused medications create opportunities for nonprescribed use and drug sharing among friends and family members, who may perceive these medications to be low risk given their storage at home.” Almost 50% of adolescents who misuse prescription opioids report getting them from friends or relatives.^11^

Understanding the parental origins of adolescent prescription opioid medical use and misuse has important public health implications, because adolescent prescription opioid use is associated with increased morbidity and mortality. Youths who use prescription opioids are more likely to be diagnosed with an opioid use disorder at 25 years of age^12^ and to experience substance-related morbidity, pharmacotherapy for alcohol use disorder, conviction for a substance-related crime,^13^ suicide,^14^ overdose, and death.^8,10,15,16^

To our knowledge, no study to date has examined the associations of medical prescription opioid use and misuse between parents and adolescents in the US. To address this gap, we examined these associations in nationally representative samples of parent-adolescent dyads, considering other characteristics of parents and adolescents that could account for the observed associations in prescription opioid use. We addressed 2 questions: (1) What are the associations between parental and adolescent prescription opioid medical use and misuse? and (2) What other parental and adolescent factors are associated with adolescent prescription opioid medical use and misuse? We hypothesized that parental medical prescription opioid use would be associated with adolescent medical prescription opioid use and misuse, whereas parental prescription opioid misuse would be associated only with adolescent prescription opioid misuse. We also hypothesized that negative quality of the parent-adolescent relationship and adolescent depression and delinquency would be associated with adolescent prescription opioid misuse.

## Methods

### Participants and Procedures

Data are from the 2015-2017 NSDUH annual, nationally representative cross-sectional surveys of the US population 12 years and older.^17^ The target civilian noninstitutionalized population represents more than 98% of the US population. Persons living in noninstitutional group quarters (homeless shelters, rooming houses, or college dormitories) and civilians living on military bases are included; individuals on active military duty, in jail, in drug treatment programs, and in hospitals and homeless persons not in shelters are excluded. Approximately 67 500 persons are interviewed in person annually. Substance use and sensitive behaviors are ascertained through audio computer-assisted self-interviewing. Completion rates are 50% to 55%. The New York State Psychiatric Institute–Columbia University Department of Psychiatry institutional review board approved the study, which implemented secondary data analysis of restricted-use NSDUH data at a Federal Statistical Research Data Center. Because of coronavirus disease 2019–related circumstances, we could not access the restricted data to implement new analyses in response to reviewers’ questions. Instead, we implemented analyses on the public-use 2015-2017 data on respondents who would approximate parents and adolescent offspring in the analytical sample, namely, adults 30 years and older living with children younger than 18 years and youths aged 12 to 17 years living with a mother or father. The NSDUH obtained written informed consent from adult respondents 18 years and older and from parents/guardians for respondents aged 12 to 17 years. This study followed the Strengthening the Reporting of Observational Studies in Epidemiology (STROBE) reporting guideline (eMethods 2 in the Supplement indicates the percentages missing and how missing data were addressed in the model estimation).

In selected households, the NSDUH selects 2 persons to be interviewed based on a pair-sampling algorithm.^18,19^ We identified analytical samples of 15 200 parent-child dyads with an adolescent aged 12 to 17 years (9400 mothers and 5800 fathers), weighted to be nationally representative of adolescents living with their mother or father.^4^ Selection and weighting procedures are described in eMethods 1 in the Supplement.

### Measures

#### Prescription Opioid Medical Use and Misuse

Data were collected from January 6, 2015, to December 20, 2017. Parent and adolescent past 12-month prescription opioid misuse and medical use was defined as per the NSDUH based on 2 questions. A screener asked about the use of 37 (in 2015) or 36 (in 2016 and 2017) prescription opioids in the past 12 months, grouped into 12 classes (eTable 1 in the Supplement). Respondents were then asked about having misused each selected prescription opioid as “using the drug in any way not directed by a doctor, including without a prescription of one’s own; in greater amounts, more often, or longer than prescribed; any other way.” A positive answer for any drug defined the respondent as a misuser; medical use was having used exclusively without any misuse in the past year. Medical use and misuse were defined as mutually exclusive categories, although misusers of one drug could have also used another without misuse. The prescription opioid use variable was coded 0 for no prescription opioid use, 1 for medical prescription opioid use only, and 2 for prescription opioid misuse.

#### Covariates

Factors previously found to be associated with adult and adolescent prescription opioid medical use and misuse^5,20,21,22,23,24,25,26,27,28,29,30,31,32,33,34,35,36,37^ were included as covariates in the models. These variables, defined in eMethods 2 in the Supplement, included parental past 12-month use of prescription benzodiazepines, stimulants, tobacco, alcohol, marijuana, and other illicit drugs; parents’ and adolescents’ perceived harm due to drug use, depression, delinquency, and physical health; adolescents’ report of the quality of the parent-adolescent relationship, perceived schoolmates’ drug use, and religiosity; and sociodemographic characteristics (age, sex, race/ethnicity, parental educational level, marital status, and residential population density).

### Statistical Analysis

Data were analyzed from October 4, 2019, to October 15, 2020. We examined the prevalence of past 12-month prescription opioid medical use and misuse among parents and adolescents in dyads overall and by sex, race/ethnicity, and child age. Then we examined the bivariate associations between parental and adolescent medical prescription opioid use and misuse. To determine the unique associations between parent and adolescent prescription opioid use and misuse, we implemented multivariable multinomial logistic regression analyses, controlling for parental other drug use and parent and adolescent psychosocial, health, and sociodemographic characteristics. The outcome was the 3-category adolescent prescription opioid use variable: medical use only, misuse, and no use (reference group). The model estimated the odds of an adolescent being a prescription opioid medical user or misuser compared with being a nonuser for each characteristic. Three 2-way interactions (parental prescription opioid use by parent sex, by child sex, and by child race/ethnicity) were estimated to determine whether associations between parental and adolescent medical prescription opioid use and misuse differed by these characteristics. Statistical significance was determined using 2-sided *t* tests with a significance level at *P* < .05.

All analyses were implemented in SUDAAN, version 11.0.1 (RTI International),^38^ with design effects adjusted by a Taylor series linearization and sample weights reflecting selection probabilities at various stages of the sampling design. Pair-level weights provided by NSDUH were used.^18,19^

## Results

### Sample Characteristics

Table 1 presents characteristics of parents and adolescents in the sample of 15 200 parent-adolescent dyads. Among parents, mean (SD) age was 44.3 (7.4) years; 56.6% were mothers, and 43.4% were fathers; and 76.0% were married. Among adolescents, mean (SD) age was 14.5 (1.7) years; 47.2% were girls, and 52.8% were boys. In regard to race/ethnicity, 54.7% of adolescents were non-Hispanic White, 11.3% were non-Hispanic African American, 24.0% were Hispanic, and 10.0% were other.

**Table 1.** Characteristics of Parents and Adolescents in Parent-Adolescent Dyads^a^

Characteristic	Parent (n = 15 200)	Adolescent (n = 15 200)
Age, mean (SD), y	44.3 (7.4)	14.5 (1.7)
Female	56.6	47.2
Race/ethnicity		
White	58.6	54.7
African American	11.5	11.3
Hispanic	21.2	24.0
Other^b^	8.7	10.0
Educational level		
High school graduate or less	34.9	NA
Some college	29.9	NA
College graduate	35.2	NA
Marital status		
Married	76.0	NA
Separated or divorced	13.5	NA
Widowed	1.4	NA
Never married	9.1	NA
Population density		
Not in CBSA	5.6	NA
CBSA		
<1 Million persons	37.7	NA
≥1 Million persons	56.7	NA
Substance use in past 12 mo		
Smoking	20.7	NA
Alcohol	71.7	NA
Marijuana	8.5	NA
Other illicit drugs^c^	0.9	NA
Prescription opioids^d^		
Medical use only	31.5	15.7
Misuse	4.1	3.2
Prescription benzodiazepines		
Medical use only	10.0	NA
Misuse	1.0	NA
Prescription stimulants		
Medical use only	4.8	NA
Misuse	0.7	NA
Perceived harm of drug use, mean (SD)^e^	4.6 (1.4)	4.0 (1.7)
Perceived parenting in past 12 mo, mean (SD)		
Lack of monitoring^f^	NA	2.1 (0.7)
Lack of support^f^	NA	1.6 (0.8)
Conflict^g^	NA	2.8 (1.4)
Religiosity, mean (SD)^f^	NA	2.6 (0.9)
Perceived drug use of schoolmates^h^		
None or few	NA	63.9
Most or all	NA	36.1
Delinquency in past 12 mo, mean (SD)^i^	0.02 (0.2)	0.4 (0.8)
Depression in past 12 mo	5.5	12.4
Health conditions (lifetime)		
1	23.7	15.7
≥2	8.5	1.2
Perceived health		
Excellent, very good, or good	89.1	96.1
Fair or poor	10.9	3.9

Abbreviations: CBSA, core-based statistical area; NA, not applicable.

^a^ Data are from the 2015-2017 National Survey on Drug Use and Health. Unless otherwise indicated, data are expressed as weighted percentages from unweighted numbers based on sample sizes of greater than 100.

^b^ Includes Asian, Native American/Alaska Native, Native Hawaiian/Other Pacific Islander, and more than 1 race.

^c^ Includes cocaine, heroin, and hallucinogens.

^d^ See eTable 1 in the Supplement for the specific prescription opioid pain relievers asked about.

^e^ Scores range from 0 to 6, with higher scores indicating higher levels of the variable. See eMethods 2 in the Supplement for the specific definitions.

^f^ Scores range from 1 to 4, with higher scores indicating higher levels of the variable. See eMethods 2 in the Supplement for the specific definitions.

^g^ Scores range from 1 to 5, with higher scores indicating higher levels of the variable. See eMethods 2 in the Supplement for the specific definitions.

^h^ Includes smoking, alcohol, and marijuana.

^i^ Scores range from 0 to 3 for parents and 0 to 6 for adolescents, with higher scores indicating higher levels of the variable. See eMethods 2 in the Supplement for the specific definitions.

### Prescription Opioid Medical Use and Misuse Among Parents and Adolescents

Almost 36% of parents used prescription opioids in the past 12 months: 31.5% (95% CI, 30.3%-32.7%) used only medically as prescribed, and 4.1% (95% CI, 3.6%-4.6%) misused (Table 2). Mothers had higher rates of medical use than fathers (33.5% [95% CI, 32.0%-35.1%] vs 28.9% [95% CI, 27.0%-30.8%]) but similar rates of misuse (3.8% [95% CI, 3.2%-4.5%] vs 4.4% [95% CI, 3.7%-5.3%]). African American parents had higher rates of medical use (39.2% [95% CI, 35.5%-43.2%]) but lower rates of misuse (2.5% [95% CI, 1.8%-3.5%]) than White (33.8% [95% CI, 32.1%-35.4%] and 4.3% [3.7%-5.0%], respectively) and Hispanic (24.1% [95% CI, 2.18%-26.6%] and 4.7% [95% CI, 3.6%-6.0%], respectively) parents.

**Table 2.** Prevalence of Past 12-Month Medical Prescription Opioid Use and Misuse Among Parent-Adolescent Dyads by Sociodemographic Characteristics

Respondent	Prescription opioid use group, % (95% CI)^a^		Wald *F* test value (*df*)
	Medical use only	Misuse	
**Parent**			
All	31.5 (30.3-32.7)	4.1 (3.6-4.6)	NA
Sex			
Female	33.5 (32.0-35.1)^b^	3.8 (3.2-4.5)	7.2 (2)
Male	28.9 (27.0-30.8)	4.4 (3.7-5.3)	
Race/ethnicity			
White	33.8 (32.1-35.4)^c^	4.3 (3.7-5.0)^d^	12.9 (6)
African American	39.2 (35.5-43.2)	2.5 (1.8-3.5)	
Hispanic	24.1 (21.8-26.6)	4.7 (3.6-6.0)	
Other^e^	23.9 (20.2-28.2)	3.2 (2.0-5.1)	
**Adolescent**			
All	15.7 (14.8-16.7)	3.2 (2.7-3.7)	NA
Sex			
Female	17.1 (15.7-18.5)^b^	3.1 (2.6-3.8)	3.8 (2)
Male	14.4 (13.2-15.8)	3.2 (2.5-4.0)	
Race/ethnicity			
White	15.8 (14.6-17.2)	3.2 (2.5-4.0)	1.8 (6)
African American	17.1 (14.5-20.0)	4.1 (2.9-5.8)	
Hispanic	15.3 (13.5-17.2)	3.1 (2.3-4.2)	
Other	14.2 (11.3-17.7)	2.0 (1.3-3.1)	
Age, y			
12	9.7 (8.2-11.5)^f^	1.2 (0.7-2.1)^g^	10.7 (10)
13	12.6 (10.7-14.7)	2.0 (1.4-2.9)	
14	15.6 (13.4-18.1)	2.5 (1.7-3.7)	
15	18.0 (15.6-20.6)	3.8 (2.5-5.8)	
16	18.7 (16.0-21.6)	4.0 (2.9-5.5)	
17	19.5 (17.0-22.3)	5.5 (4.1-7.2)	

Abbreviation: NA, not applicable.

^a^ Data are from the 2015-2017 National Survey on Drug Use and Health (n = 15 200). The weighted percentages for each level of the categorical variables are based on sample sizes (unweighted numbers) of greater than 100.

^b^ Differences were significant by groups for medical use (*P* < .05) using 2-sided *t* tests.

^c^ Differences were significant by race/ethnicity groups for medical use (*P* < .05) using 2-sided *t* tests. White was different compared with all other race/ethnicity groups; African American was different compared with all other race/ethnicity groups.

^d^ Differences were significant by race/ethnicity groups for misuse (*P* < .05) using 2-sided *t* tests. White was different compared with African American. African American was different compared with Hispanic.

^e^ Includes Asian, Native American/Alaska Native, Native Hawaiian/Other Pacific Islander, and more than 1 race.

^f^ Differences were significant by age groups for medical use (*P* < .05) using 2-sided *t* tests. Age 12 was different compared with all other age groups. Age 13 was different compared with all other age groups. Age 14 was different compared with age 17.

^g^ Differences were significant by age groups for misuse (*P* < .05) using 2-sided *t* tests. Age 12 was different compared with age groups 14, 15, 16, and 17. Age 13 was different compared with age groups 15, 16, and 17. Age 14 was different compared with age groups 16 and 17.

Almost 19% of adolescents had used prescription opioids in the past 12 months: 15.7% (95% CI, 14.8%-16.7%) used only medically as prescribed, and 3.2% (95% CI, 2.7%-3.7%) misused. The proportion of misusers among all prescription opioid users was almost 50% higher among adolescents than parents (16.9% [95% CI, 15.8%-18.1%] vs 11.5% [95% CI, 10.7%-12.4%]). Girls had higher rates of medical use than boys (17.1% [95% CI, 15.7%-18.5%] vs 14.4% [95% CI, 13.2%-15.8%]) but similar rates of misuse (3.1% [95% CI, 2.6%-3.8%] vs 3.2% [95% CI, 2.5%-4.0%]). Rates of adolescent medical use and misuse were similar across racial/ethnic groups. Prescription opioid use increased with adolescent age: medical use doubled from 9.7% (95% CI, 8.2%-11.5%) at 12 years of age to 19.5% (95% CI, 17.0%-22.3%) at 17 years of age; misuse quintupled from 1.2% (95% CI, 0.7%-2.1%) to 5.5% (95% CI, 4.1%-7.2%).

The NSDUH does not report rates of concurrent prescription opioid misuse and medical use. Some crude estimates can be made from the answers to 2 questions: one about the overall way of misusing opioids and another about the source of the last prescription opioid misuse, in which the first 2 alternatives are a physician’s prescription (eTable 2 in the Supplement). We estimated that, in the 2015-2017 public data among adults living with children younger than 18 years, 59.7% of misusers misused their own prescriptions, and 66.0% misused without a prescription. Among adolescents living with a mother or father, the percentages were 48.6% and 73.0%, respectively (eTable 2 in the Supplement).

### Associations Between Parent and Adolescent Prescription Opioid Medical Use and Misuse

There were significant and positive bivariate associations between past 12-month prescription opioid use by parents and adolescents; the associations differed by type of parental use. Parental exclusive medical use was associated with higher rates of adolescent exclusive medical use and misuse, whereas parental misuse was associated only with a higher rate of adolescent misuse. When a parent had used prescription opioids only medically, 18.6% (95% CI, 16.8%-20.6%) of adolescents also used medically compared with 14.3% (95% CI, 10.9%-18.4%) when a parent misused prescription opioids and 14.3% (95% CI, 13.2%-15.5%) when a parent had not used prescription opioids. Compared with when parents had not used any prescription opioids, the unadjusted odds ratio (OR) of adolescents’ medical use was 1.40 (95% CI, 1.20-1.64) when parents used only medically and 1.03 (95% CI, 0.72-1.48) when parents misused. The proportions of adolescent misusers were 4.1% (95% CI, 3.2%-5.3%) when parents only used medically, 4.9% (95% CI, 3.0%-7.7%) when parents misused, and 2.6% (95% CI, 2.1%-3.1%) when parents had not used. Compared with when parents had not used prescription opioids, the ORs for adolescent prescription opioid misuse were 1.73 (95% CI, 1.26-2.38) when parents only used medically and 1.94 (95% CI, 1.10-3.44) when parents misused (Table 3).

**Table 3.** Parent and Adolescent Factors Associated With Adolescent Past 12-Month Medical Prescription Opioid Use and Misuse

Respondent characteristic	Adolescent past 12-mo prescription opioid use^a^			
	Bivariate model, OR (95% CI)		Multivariable model, aOR (95% CI)	
	Medical use only vs no use	Misuse vs no use	Medical use only vs no use	Misuse vs no use
**Parent**				
Age, y	1.01 (1.00-1.02)	0.97 (0.95-1.00)	1.01 (0.99-1.02)	0.97 (0.95-1.00)
Mothers (vs fathers)	0.96 (0.82-1.12)	1.22 (0.88-1.69)	0.95 (0.80-1.12)	1.02 (0.68-1.55)
Educational level				
College graduate	1 [Reference]	1 [Reference]	1 [Reference]	1 [Reference]
Some college	1.13 (0.94-1.36)	1.62 (1.08-2.43)	0.99 (0.80-1.21)	1.00 (0.65-1.55)
High school graduate or less	1.15 (0.96-1.38)	1.50 (1.01-2.22)	1.02 (0.81-1.28)	1.02 (0.62-1.67)
Marital status				
Married	1 [Reference]	1 [Reference]	1 [Reference]	1 [Reference]
Widowed	1.03 (0.60-1.78)	2.05 (0.70-6.02)	0.84 (0.47-1.48)	1.94 (0.55-6.89)
Separated or divorced	1.06 (0.87-1.29)	1.55 (1.00-2.39)	0.88 (0.71-1.09)	1.06 (0.62-1.82)
Never married	1.19 (0.97-1.46)	1.99 (1.34-2.97)	1.10 (0.86-1.40)	1.48 (0.92-2.39)
Population density				
Not in CBSA	1 [Reference]	1 [Reference]	1 [Reference]	1 [Reference]
CBSA				
<1 Million persons	0.77 (0.58-1.01)	0.79 (0.49-1.29)	0.73 (0.54-0.98)	0.65 (0.39-1.09)
≥1 Million persons	0.62 (0.47-0.82)	0.67 (0.41-1.10)	0.60 (0.44-0.81)	0.59 (0.33-1.05)
Substance use in past 12 mo				
None	1 [Reference]	1 [Reference]	1 [Reference]	1 [Reference]
Smoking	1.20 (1.02-1.42)	1.70 (1.25-2.31)	1.05 (0.85-1.28)	1.02 (0.70-1.49)
Alcohol	1.08 (0.92-1.28)	1.25 (0.91-1.72)	1.05 (0.87-1.26)	1.06 (0.73-1.53)
Marijuana	1.23 (0.93-1.64)	2.37 (1.51-3.73)	1.13 (0.84-1.54)	1.84 (1.13-2.99)
Other illicit drugs^b^	0.87 (0.37-2.05)	5.52 (1.57-19.36)	0.59 (0.20-1.76)	3.31 (0.55-19.85)
Prescription opioids				
Medical use only	1.40 (1.20-1.64)	1.73 (1.26-2.38)	1.28 (1.06-1.53)	1.53 (1.07-2.25)
Misuse	1.03 (0.72-1.48)	1.94 (1.10-3.44)	0.86 (0.56-1.32)	0.94 (0.51-1.76)
Prescription benzodiazepines				
Medical use only	1.38 (1.12-1.69)	1.79 (1.09-2.95)	1.23 (0.97-1.56)	1.43 (0.83-2.48)
Misuse	0.79 (0.36-1.72)	2.09 (0.90-4.85)	0.77 (0.33-1.79)	1.67 (0.69-4.06)
Prescription stimulants				
Medical use only	1.55 (1.13-2.11)	1.58 (0.93-2.68)	1.40 (1.02-1.91)	1.06 (0.59-1.88)
Misuse	1.05 (0.60-1.81)	0.48 (0.13-1.86)	1.15 (0.65-2.04)	0.23 (0.05-1.08)
Perceived harm of drug use^c^	0.91 (0.85-0.98)	0.95 (0.84-1.07)	0.93 (0.86-1.02)	1.09 (0.92-1.29)
Delinquency in past 12 mo^c^	1.04 (0.99-1.10)	1.00 (0.91-1.10)	1.04 (0.98-1.10)	0.88 (0.76-1.03)
Depression in past 12 mo				
No	1 [Reference]	1 [Reference]	1 [Reference]	1 [Reference]
Yes	1.35 (1.04-1.76)	1.41 (0.87-2.28)	1.17 (0.88-1.58)	1.00 (0.58-1.73)
Health conditions (lifetime)				
None	1 [Reference]	1 [Reference]	1 [Reference]	1 [Reference]
1	1.18 (0.99-1.41)	1.07 (0.75-1.53)	1.11 (0.92-1.33)	1.06 (0.71-1.59)
≥2	1.36 (1.04-1.78)	0.98 (0.63-1.53)	1.15 (0.86-1.54)	0.70 (0.41-1.20)
Perceived health as fair or poor (vs excellent to good)	1.08 (0.87-1.33)	1.11 (0.76-1.61)	0.83 (0.65-1.07)	0.76 (0.49-1.20)
**Adolescent**				
Age	1.18 (1.13-1.23)	1.36 (1.24-1.48)	1.09 (1.03-1.15)	1.15 (1.04-1.28)
Girls (vs boys)	1.22 (1.06-1.41)	1.01 (0.76-1.35)	1.08 (0.92-1.26)	0.93 (0.66-1.29)
Race/ethnicity				
White	1 [Reference]	1 [Reference]	1 [Reference]	1 [Reference]
African American	1.11 (0.89-1.38)	1.36 (0.89-2.08)	1.09 (0.83-1.43)	1.00 (0.56-1.78)
Hispanic	0.96 (0.81-1.14)	0.99 (0.67-1.47)	1.09 (0.88-1.34)	0.92 (0.56-1.52)
Other^d^	0.87 (0.65-1.15)	0.61 (0.37-1.01)	1.04 (0.77-1.39)	0.78 (0.46-1.32)
Perceived parenting in past 12 mo^c^				
Lack of monitoring	1.13 (1.05-1.21)	1.42 (1.26-1.60)	1.00 (0.92-1.10)	1.08 (0.91-1.28)
Lack of support	1.12 (1.04-1.19)	1.37 (1.20-1.55)	1.03 (0.95-1.12)	1.04 (0.88-1.24)
Conflict	1.17 (1.09-1.26)	1.59 (1.39-1.82)	1.07 (0.98-1.17)	1.26 (1.05-1.52)
Religiosity^c^	0.97 (0.90-1.04)	0.79 (0.68-0.93)	1.05 (0.97-1.13)	1.00 (0.84-1.20)
Perceived harm of drug use^c^	0.95 (0.88-1.02)	0.70 (0.63-0.77)	0.95 (0.88-1.03)	0.73 (0.64-0.83)
Perceived most or all schoolmates use drugs (vs none or few)^e^	2.03 (1.73-2.38)	4.91 (3.43-7.03)	1.61 (1.32-1.96)	2.87 (1.95-4.23)
Delinquency in past 12 mo^c^	1.20 (1.13-1.27)	1.73 (1.58-1.90)	1.13 (1.05-1.22)	1.55 (1.38-1.74)
Depression in past 12 mo				
No	1 [Reference]	1 [Reference]	1 [Reference]	1 [Reference]
Yes	1.61 (1.31-1.99)	2.92 (2.14-3.98)	1.17 (0.93-1.47)	1.75 (1.26-2.44)
Health conditions (lifetime)				
None	1 [Reference]	1 [Reference]	1 [Reference]	1 [Reference]
1	1.11 (0.93-1.34)	0.79 (0.55-1.14)	1.01 (0.83-1.23)	0.61 (0.42-0.90)
≥2	1.72 (1.04-2.85)	1.14 (0.51-2.54)	1.44 (0.86-2.42)	0.88 (0.43-1.81)
Perceived health as fair or poor (vs excellent to good)	1.37 (0.95-1.97)	1.38 (0.84-2.27)	1.32 (0.92-1.90)	1.05 (0.57-1.94)

Abbreviations: aOR, adjusted odds ratio (OR); CBSA, core-based statistical area.

^a^ Data are from the 2015-2017 National Survey on Drug Use and Health (n = 15 200). Weighted estimates are calculated from unweighted numbers. Survey year is controlled for.

^b^ Includes cocaine, heroin, and hallucinogens.

^c^ Calculated using standardized scores.

^d^ Includes Asian, Native American/Alaska Native, Native Hawaiian/Other Pacific Islander, and more than 1 race.

^e^ Includes cigarettes, alcohol, and marijuana.

Controlling for all psychosocial and demographic factors (Table 3), the associations between parental prescription opioid medical use with adolescent prescription opioid medical use (aOR, 1.28; 95% CI, 1.06-1.53) and misuse (aOR, 1.53; 95% CI, 1.07-2.25) persisted, although they were attenuated, whereas the association between parental and adolescent prescription opioid misuse was no longer significant (aOR, 0.94; 95% CI, 0.51-1.76). Parental-adolescent associations on prescription opioid use did not differ by parent or adolescent sex and race/ethnicity.

### Additional Correlates of Adolescent Prescription Opioid Medical Use and Misuse

Parental use of substances other than prescription opioids was significantly associated with adolescent medical prescription opioid use and/or misuse at the bivariate level. Parental smoking and medical prescription benzodiazepine use was associated with adolescent medical prescription opioid use (ORs, 1.20 [95% CI, 1.02-1.42] and 1.38 [95% CI, 1.12-1.69], respectively) and misuse (ORs, 1.70 [95% CI, 1.25-2.31] and 1.79 [95% CI, 1.09-2.95], respectively); parental medical prescription stimulant use was associated with adolescent medical prescription opioid use (OR, 1.55 [95% CI, 1.13-2.11]); and parental use of marijuana and other illicit drugs was associated with adolescent prescription opioid misuse (ORs, 2.37 [95% CI, 1.51-3.73] and 5.52 [95% CI, 1.57-19.36], respectively) (Table 3). With control for all risk factors and parental use of drugs other than prescription opioids, parental medical stimulant use remained uniquely associated with adolescent medical prescription opioid use (aOR, 1.40; 95% CI, 1.02-1.91) and parental marijuana use with adolescent prescription opioid misuse (aOR, 1.84; 95% CI, 1.13-2.99).

Although adolescent perceived quality of parenting was associated with higher rates of adolescent medical prescription opioid use (OR for low level of monitoring, 1.13 [95% CI, 1.05-1.21]; OR for low level of support, 1.12 [95% CI, 1.04-1.19]; and OR for high level of parent-adolescent conflict, 1.17 [95% CI, 1.09-1.26]) and, especially, misuse at the bivariate level (OR for low level of monitoring, 1.42 [95% CI, 1.26-1.60]; OR for low level of support, 1.37 [95% CI, 1.20-1.55]; and OR for high level of parent-adolescent conflict, 1.59 [95% CI, 1.39-1.82]), after controlling for other factors, only parent-adolescent conflict retained a unique association with adolescent prescription opioid misuse (aOR, 1.26; 95% CI, 1.05-1.52). Bivariate associations of parental and adolescent health conditions with adolescent medical prescription opioid use did not persist in the multivariable models. In these models, adolescents who engaged in delinquent behavior and perceived that most of their schoolmates used drugs were more likely to use prescription opioids medically and to misuse than other adolescents, with stronger associations for misuse than medical use (delinquency aORs, 1.55 [95% CI, 1.38-1.74] vs 1.13 [95% CI, 1.05-1.22]; schoolmate drug use aORs, 2.87 [95% CI, 1.95-4.23] vs 1.61 [95% CI, 1.32-1.96]). Adolescent depression was associated only with prescription opioid misuse (aOR, 1.75; 95% CI, 1.26-2.44). Adolescents who believed that drug use was harmful were less likely to misuse prescription opioids. Adolescent age was positively associated with prescription opioid medical use and misuse (aORs, 1.09 [95% CI, 1.03-1.15] and 1.15 [95% CI, 1.04-1.28], respectively); race/ethnicity was not. Adolescents living in metropolitan areas had a lower rate of medical prescription opioid use than those in nonmetropolitan areas (aORs, 0.73 [95% CI, 0.54-0.98] and 0.60 [95% CI, 0.44-0.81], respectively).

### Characteristics of Parents Who Use Opioids Medically vs Those Who Misuse Prescription Opioids

We compared the substance use, mental health, and sociodemographic characteristics of parental prescription opioid misusers with exclusive medical users and nonusers. Prescription opioid misusers, and to a lesser extent exclusive medical users, used drugs other than prescription opioids more than nonusers. Prescription opioid misusers had higher rates of smoking, and especially marijuana and other illicit drug use, and higher rates of benzodiazepine and stimulant misuse than medical users and, especially, nonusers. Thus, 24.0% (95% CI, 19.6%-29.0%) of parental prescription opioid misusers had used marijuana in the past 12 months compared with 11.3% (95% CI, 9.9%-13.0%) of medical prescription opioid users and 6.1% (95% CI, 5.4%-6.8%) of nonusers; 10.9% (95% CI, 7.9%-14.8%) of prescription opioid misusers, 0.9% (95% CI, 0.6%-1.4%) of medical prescription opioid users, and 0.4% (95% CI, 0.2%-0.5%) of nonusers had misused benzodiazepines (Table 4). Parental prescription opioid misusers and medical users were equally likely to drink alcohol (77.0% [95% CI, 71.7%-81.5%] and 74.8% [95% CI, 72.7%-76.9%], respectively) and to have used benzodiazepines medically (18.8% [95% CI, 14.2%-24.6%] and 17.5% [95% CI, 15.8%-19.2%], respectively) and stimulants (9.4% [95% CI, 6.6%-13.1%] and 8.0% [95% CI, 6.9%-9.2%], respectively). Compared with parents who had not used prescription opioids and parental medical users, parental misusers were more likely to be less educated (high school graduate or less, 43.0% [95% CI, 37.2%-49.1%] vs 35.1% [95% CI, 33.5%-36.8%] and 33.6% [95% CI, 31.5-35.7]), never married (16.2% [95% CI, 12.5%-20.7%] vs 8.3% [95% CI, 7.5%-9.2%] and 9.9% [95% CI, 8.8-11.2]), more depressed (13.2% [95% CI, 10.0%-17.3%] vs 3.6% [95% CI, 3.1%-4.1%] and 8.6% [95% CI, 7.5-9.9]), and more delinquent (score range, 0-3; mean [SD], 0.07 [0.30] vs 0.01 [0.20] and 0.02 [0.2]). Medical users were more depressed than nonusers (8.6% [95% CI, 7.5%-9.9%] vs 3.6% [95% CI, 3.1%-4.1%]) but less depressed than misusers (13.2% [95% CI, 10.0%-17.3%]) and experienced more health conditions than misusers (≥2 conditions, 14.7% [95% CI, 13.0%-16.6%] vs 9.5% [95% CI, 7.1%-12.5%]), although both perceived their health to be poor (16.8% [95% CI, 15.2%-18.7%] and 16.6% [12.5%-21.7%], respectively).

**Table 4.** Characteristics of Parents by Past 12-Month Medical Prescription Opioid Use and Misuse

Characteristic	Parent past 12-mo prescription opioid use^a^			Wald *F* test value (*df*)^b^
	No prescription opioid use	Medical use only	Prescription opioid misuse	
Female	55.1 (53.5-56.7)	60.2 (57.9-62.5)^c^	52.8 (46.7-58.8)	7.0 (2)
Educational level				
High school graduate or less	35.1 (33.5-36.8)^d^	33.6 (31.5-35.7)^d^	43.0 (37.2-49.1)	13.9 (4)
Some college	27.2 (25.7-28.6)^e^	35.2 (33.0-37.4)	31.9 (26.4-37.8)	
College graduate	37.7 (36.0-39.5)^f^	31.2 (29.1-33.5)^d^	25.1 (20.2-30.8)	
Marital status				
Married	78.8 (77.6-80.0)^f^	71.3 (69.2-73.4)	67.7 (61.9-73.0)	10.3 (6)
Separated or divorced	11.5 (10.6-12.5)^e^	17.2 (15.5-19.0)	15.0 (11.1-19.9)	
Widowed	1.4 (1.0-1.8)	1.6 (1.2-2.3)	1.1 (0.5-2.5)	
Never married	8.3 (7.5-9.2)^f^	9.9 (8.8-11.2)^d^	16.2 (12.5-20.7)	
Population density				
Not in CBSA	5.2 (4.5-6.0)	6.3 (5.3-7.6)	5.9 (3.4-9.9)	6.0 (4)
CBSA				
<1 Million persons	35.6 (34.0-37.3)^e^	41.5 (39.2-43.8)	41.0 (35.1-47.0)	
≥1 Million persons	59.2 (57.5-60.9)^e^	52.2 (49.8-54.6)	53.1 (47.0-59.3)	
Substance use in past 12 mo				
Smoking	17.2 (16.0-18.4)^f^	26.5 (24.7-28.4)^d^	32.3 (27.1-38.0)	48.9 (2)
Alcohol	69.8 (68.1-71.4)^f^	74.8 (72.7-76.9)	77.0 (71.7-81.5)	8.5 (2)
Marijuana	6.1 (5.4-6.8)^f^	11.3 (9.9-13.0)^d^	24.0 (19.6-29.0)	67.3 (2)
Other illicit drugs^g^	0.4 (0.3-0.6)^f^	1.1 (0.6-1.9)^d^	5.8 (4.0-8.4)	40.9 (2)
Prescription benzodiazepines				
Medical use only	5.7 (5.0-6.6)^f^	17.5 (15.8-19.2)	18.8 (14.2-24.6)	96.5 (4)
Misuse	0.4 (0.2-0.5)^f^	0.9 (0.6-1.4)^d^	10.9 (7.9-14.8)	
Prescription stimulants				
Medical use only	3.0 (2.5-3.5)^f^	8.0 (6.9-9.2)	9.4 (6.6-13.1)	42.1 (4)
Misuse	0.4 (0.2-0.6)^f^	0.8 (0.5-1.3)^d^	5.1 (3.4-7.6)	
Perceived harm of drug use, mean (SD)^h^	4.7 (1.4)^f^	4.5 (1.3)^d^	4.2 (1.5)	30.7 (2)
Depression in past 12 mo	3.6 (3.1-4.1)^f^	8.6 (7.5-9.9)^d^	13.2 (10.0-17.3)	25.1 (4)
Delinquency in past 12 mo, mean (SD)^i^	0.01 (0.2)^d^	0.02 (0.2)^d^	0.07 (0.3)	12.0 (2)
Health conditions (lifetime)				
1	21.7 (20.4-23.1)^e^	27.9 (25.9-29.9)	23.7 (19.2-28.9)	45.5 (4)
≥2	5.5 (4.7-6.0)^f^	14.7 (13.0-16.6)^d^	9.5 (7.1-12.5)	
Perceived health as fair or poor	7.6 (6.8-8.5)^f^	16.8 (15.2-18.7)	16.6 (12.5-21.7)	57.2 (2)

Abbreviation: CBSA, core-based statistical area.

^a^ Data are from the 2015-2017 National Survey on Drug Use and Health (n = 15 200). Unless otherwise indicated, data are expressed as weighted percentages from unweighted numbers based on sample sizes of greater than 100.

^b^ Based on multinomial logistic regression of each characteristic on parental prescription opioid use.

^c^ Differences were significant compared with groups with no use and misuse (*P* < .05) using 2-sided *t* tests.

^d^ Differences were significant compared with the group with misuse (*P* < .05) using 2-sided *t* tests.

^e^ Differences were significant compared with the group with medical use only (*P* < .05) using 2-sided *t* tests.

^f^ Differences were significant compared with groups with medical use only and misuse (*P* < .05) using 2-sided *t* tests.

^g^ Includes cocaine, heroin, and hallucinogens.

^h^ Scores range from 0 to 6, with higher scores indicating higher levels of the variable. See eMethods 2 in the Supplement for the specific definitions.

^i^ Scores range from 0 to 3, with higher scores indicating higher levels of the variable. See eMethods 2 in the Supplement for the specific definitions.

## Discussion

Parental use of prescription opioids in the US is extremely high and an important source of prescription opioid exposure for children. In this large national sample of parent-adolescent dyads surveyed from 2015 to 2017, 1 in 3 parents and 1 in 6 adolescents used a prescription opioid in the past 12 months: 31.5% of parents used only medically and 4.1% misused, whereas 15.7% of adolescents used only medically and 3.2% misused.

The most noteworthy finding of the study is that, after controlling for other factors, parental exclusive past 12-month medical prescription opioid use was associated both with adolescent medical prescription opioid use and misuse, whereas parental prescription opioid misuse was not associated with either. Without control for other factors, parental prescription opioid misuse was associated only with adolescent misuse. With control for key variables, including parental use and misuse of other drugs, adolescent perceived quality of parenting and perceived schoolmates’ drug use, parental and adolescent depression, delinquency, perceived harm due to drug use, and health conditions, the association between parental and adolescent prescription opioid misuse did not persist. The univariate association between parental and offspring prescription opioid misuse included the effect of parental use of other drugs, in particular marijuana. The offspring of parent prescription opioid misusers were disproportionately exposed to parental polysubstance misuse. Thus, parental use of other drugs could partially account for the bivariate association between parent and adolescent prescription opioid misuse. It should be noted that, in years 2004 to 2012, we found an association between parental and adolescent lifetime prescription opioid misuse (aOR, 1.30 [95% CI, 1.09-1.56]).^5^ A focus on lifetime rather than past 12-month misuse and other methodological and historical differences between the 2 studies may account for differences in findings.

After controlling for parental use of other drugs and other covariates, parental marijuana use remained associated with adolescent prescription opioid misuse. By contrast, a recent study^4^ reported no multivariate association of parental marijuana use with adolescent opioid misuse in the NSDUH. The discrepancy in findings may be accounted for by the inclusion of heroin in the definition of adolescent opioid misuse in that study but not the present one. The association of parental marijuana use with adolescent prescription opioid misuse may reflect direct effects of parental marijuana use on adolescent prescription opioid use and the higher use of marijuana and other drugs by adolescent prescription opioid misusers compared with medical users.^20^ Because the 12-month measures of drug use in the NSDUH provide no information on the timing of use of these drugs in association with prescription opioid use, they were not included in the models.

The present findings highlight the important association between parental prescription opioid medical use and offspring medical use and misuse. Processes that were not measured may also be relevant. Heritability may account for familial concordance on health conditions, particularly chronic pain, and psychiatric and substance use disorders comorbid with medical prescription opioid use and misuse.^6,7,39^ Family health practices, including attitudes and norms regarding medication use, especially prescription opioid use, and access to the same clinicians, may also play a role.^6,7^ The findings also illustrate the unintended consequences of prescribing practices in the US. Prescribing prescription opioids to adults, and in particular parents, is associated with increased risk of misuse by offspring. A hopeful trend is the decreasing percentages of unique individuals prescribed opioids in the US since 2015^40,41^ and the implementation of prescription drug monitoring programs.^42^

The association of parental medical prescription opioid use with adolescent prescription opioid misuse suggests that role modeling and availability of parents’ opioid medications in the household are significant familial risk factors for prescription opioid misuse among young people, although adolescents also misuse their own prescription opioid medications and prescription opioids from nonfamilial sources. Among adolescent misusers, 25.9% obtained their last misused prescription opioid from 1 or more physicians; 58.4%, from friends or relatives; and 15.7%, from other sources, including stealing from a physician’s office, buying from a drug dealer, or some other way.^11,24,26^ Thus, adolescents’ own opioid prescriptions as well as prescription opioids from other sources, including parents, contribute to availability. Adolescent prescription opioid misusers and, to a lesser extent, medical users experienced elevated rates of psychosocial risk. Adolescents with mental health comorbidities are more likely to be prescribed an opioid, to use opioids long term, and to misuse,^20,21,27,29,31^ underscoring the need to monitor closely the functioning of adolescents who are prescribed opioids.

In this historical period of increasing legalization of recreational adult marijuana use in the US, the finding of increased prescription opioid misuse by the offspring of marijuana-using parents may portend an unintended negative consequence of drug policies. Legalization is associated with increases in marijuana use and disorder among adults and possibly adolescents.^43,44,45,46^ Legalization may lead to increased adolescent marijuana use through parental marijuana use^47^ given the association between parent and offspring marijuana use.^3,4^ Legalization may also be associated with increased offspring prescription opioid misuse. Indeed, among adults, marijuana use is associated with incipient prescription opioid misuse and disorder^48^ and, as we have reported, precedes the onset of prescription opioid misuse.^49^ The observed association of parental marijuana use and offspring prescription opioid misuse reproduces patterns at the intergenerational level observed among adults.

### Limitations

This study has limitations. Only 1 parent and 1 adolescent were assessed per household, precluding the determination of the relative associations of adolescents’ prescription opioid use with mothers’ and fathers’ prescription opioid use. Self-reports of medical use were not corroborated with administrative prescription records. Genetic factors were not ascertained, nor was pain level, an important contributor to prescription opioid use and misuse. Adolescent use of opioids other than prescription opioids could not be examined, because the prevalence of heroin use was extremely low (past 12 months, 0.03%), and illicit fentanyl use was not assessed. The data are cross-sectional and limit interpretations of processes of parental influence. Although it is unlikely that adolescent prescription opioid use influences parental use, adolescent prescription opioid misuse could affect levels of parent-child conflict and adolescent mental health. Adolescents also misuse prescription opioids obtained from sources other than parents that cannot be readily identified. The NSDUH source question covers only the last prescription opioid misuse and combines friends and unspecified relatives. In addition, the NSDUH completion rates are low. The analyses are based on 2015-2017 data and need to be repeated with more recent data. Despite these limitations, the national NSDUH dyadic data provide a unique understanding of the association between parental prescription opioid use, especially medical use, and adolescent prescription opioid use and highlight the multifactorial nature of adolescent prescription opioid use in the US.

## Conclusions

This cross-sectional study is based on nationally representative dyadic samples of parent and adolescent offspring and distinguishes whether parental prescription opioid use or misuse drives the association between parent and offspring prescription opioid use. It documents the association of environmental familial factors with adolescent prescription opioid use. We found that parental medical prescription opioid use was associated with adolescent medical prescription opioid use and misuse, whereas parental misuse was not associated with either. The findings suggest several targets for preventing adolescent prescription opioid misuse. Reducing the availability of prescription opioids in the household and potential for diversion is essential. Strategies could include limiting opioid prescribing to parents, patient educational programs emphasizing the risks of parental opioid medications to their children, and guidelines for medication use practices, including safe storage and disposal, the use of lock boxes, and collection of leftover medications.^8,50,51,52^ Assessment of parental use of medically prescribed opioids could be part of pediatric practice. Nonopioid and nonpharmacological interventions (eg, psychological and behavioral) could be preferred for treating chronic pain.^53^ Prevention efforts could also target adolescent mental health, particularly depression. Although biological factors may also contribute to the association between parent and child prescription opioid use, the study suggests that structural and environmental factors are crucial contributing factors.

## Notes

Supplement. **eMethods 1.** Sampling of Pairs in National Survey on Drug Use and Health (NSDUH), 2015-2017 **eMethods 2.** Definition of Variables in National Survey on Drug Use and Health (NSDUH), 2015-2017 **eTable 1.** Prescription Opioid Pain Relievers Asked About by Class in National Survey on Drug Use and Health, 2015-2017 **eTable 2.** Prevalence of Past 12-Month Prescription Opioid Misuse With a Prescription or Without a Prescription Among Selected Adult (n=1549) and Adolescent (n=1412) Misusers in National Survey on Drug Use and Health, 2015-2017 **eReferences.**
