# Supplementary material for: Assessment of Prescription Opioid Medical Use and Misuse Among Parents and Their Adolescent Offspring in the US
Source: JAMA Netw Open. 2021 Jan 7;4(1):e2031073. doi: 10.1001/jamanetworkopen.2020.31073 (PMC7791357; doi:10.1001/jamanetworkopen.2020.31073)
Supplement: Supplement. — eMethods 1. Sampling of Pairs in National Survey on Drug Use and Health (NSDUH), 2015-2017 eMethods 2. Definition of Variables in National Survey on Drug Use and Health (NSDUH), 2015-2017 eTable 1. Prescription Opioid Pain Relievers Asked About by Class in National Survey on Drug Use and Health, 2015-2017 eTable 2. Prevalence of Past 12-Month Prescription Opioid Misuse With a Prescription or Without a Prescription Among Selected Adult (n=1549) and Adolescent (n=1412) Misusers in National Survey on Drug Use and Health, 2015-2017 eReferences. [file jamanetwopen-e2031073-s001.pdf]

Supplementary Online Content

Griesler PC, Hu MC, Wall MM, Kandel DB. Assessment of prescription opioid medical use and misuse among parents and their adolescent offspring in the US. *JAMA Netw Open*. 2021;4(1): e2031073. doi:10.1001/jamanetworkopen.2020.31073

eMethods 1. Sampling of Pairs in National Survey on Drug Use and Health (NSDUH), 2015-2017

eMethods 2. Definition of Variables in National Survey on Drug Use and Health (NSDUH), 2015-2017

eTable 1. Prescription Opioid Pain Relievers Asked About by Class in National Survey on Drug Use and Health, 2015-2017

eTable 2. Prevalence of Past 12-Month Prescription Opioid Misuse With a Prescription or Without a Prescription Among Selected Adult (n=1549) and Adolescent (n=1412) Misusers in National Survey on Drug Use and Health, 2015-2017

eReferences.

This supplementary material has been provided by the authors to give readers additional information about their work.

©2021 Griesler PC et al. *JAMA Network Open*.

eMethods 1. Sampling of Pairs in National Survey on Drug Use and Health (NSDUH), 2015-2017

Each NSDUH annual survey between 2015 and 2017 entailed five stages of selection within each state by stratum within state: (1) census tracts, (2) census block groups, (3) segments within census block groups, (4) dwelling units within segments, and (5) persons within dwelling units. A screening interview determined the eligibility and selection of zero, one, or two persons for the interview. Any two survey-eligible persons had some nonzero chance of being selected based on age. When two persons were selected, they formed a within-dwelling unit pair. The selection involved singletons aged 12-17, 18-25, 26-34, 35-49, 50+, and pairs aged 12-17 and 12-17, 12-17 and 50+, and other combinations of the age groups.

As per SAMHSA,[1] the following method was used for selecting pairs. For a given dwelling unit, if the sum of the age-specific selection probabilities was larger than 2, two persons were selected. The individual person-selection probabilities were ratio-adjusted downward to make their sum equal to 2 for use of Brewer's method. If the sum of the age-specific selection probabilities was less than 2, then zero, one, or two persons might be selected. However, if the dwelling unit had two or more persons, it was preferred that a pair had an increased chance of being selected relative to the selection of one person. To achieve this, the individual selection probabilities were scaled upward such that their sum came close to 2 and each person-selection probability did not exceed 0.99. A modification of Brewer's method was then used to select zero, one, or two persons per dwelling unit. Dwelling units, where at least one person was selected, were counted as the selected questionnaire dwelling units (QDUs). A QDU, where two persons were selected and both had completed interviews, was considered a completed person pair.

A preliminary version of the pair weight was created for use in imputation and consisted of only the pair selection probabilities and an adjustment for nonresponse. This weight created imputation-revised versions of (1) the relationship between pair members (e.g., parent, child aged 12-17); and (2) counts of certain relationships within the dwelling unit (e.g., number of children aged 12-17 living with at least one parent). The final pair weights incorporated both (1) estimates of the number of age group pairs (e.g., ages 12-17, 35-49, etc.) obtained from all screened households, and (2) estimates of the number of certain types of pairs (e.g., parent-child, partners) obtained from all households with at least one completed interview.

©2021 Griesler PC et al. *JAMA Network Open*.

eMethods 2. Definition of Variables in National Survey on Drug Use and Health (NSDUH), 2015-2017

Other Substance Use. Parent self-reported past 12-month prescription benzodiazepine and stimulant use were each coded: 0=no use, 1=medical use only, 2=misuse. Past 12-month smoking, alcohol, marijuana, and other illicit drug use (cocaine, heroin, or hallucinogens) were each coded: 0=no, 1=yes.

Perceived Harm of Drug Use. Parents' and adolescents' perceived risk of harming themselves physically and in other ways from drug use was an index that summed dichotomized items for six drugs: smoking 1^+ cigarette packs/day, having 4-5 alcohol drinks nearly every day, and using marijuana, cocaine, LSD, and heroin each once or twice/week (Cronbach's α=0.70 parents, 0.71 adolescents).

Parent-Adolescent Relationship. Adolescent perceived quality of the parent-adolescent relationship was measured by seven items about parental (1) monitoring, (2) support, and (3) conflict with adolescent. Items were rated (1=always, 2=sometimes, 3=seldom, 4=never), except conflict measured by past 12-month frequency of arguments (1=0 times, 2=1-2, 3=3-5, 4=6-9, $5=10^+$). Monitoring was the average of four items: limit setting with peers and television, homework oversight and assistance (α=0.59); support two items: encouragement and pride (α=0.85).

Major Depressive Episode (MDE). Parent and adolescent self-reported past 12-month MDE (0=no, 1=yes) was assessed using developmentally appropriate modules based on the *Diagnostic and Statistical Manual of Mental Disorders, Fourth Edition (DSM-IV)*. MDE was defined as having at least 5 of 9 symptoms nearly every day in a 2-week period, per DSM-IV criteria.

Delinquency. Parent and adolescent self-reported past 12-month delinquency was the sum of three dichotomized items among parents [stealing something >$50, attacking with intent to hurt, and selling illegal drugs (α=0.61)], and six items among adolescents [the same 3 items as for parents, plus fighting at school/work, group fighting, and carrying a handgun (α=0.56)].

Perceived Schoolmates' Drug Use. The highest value of the adolescents' perceived same grade schoolmates' smoking, drinking or marijuana use: 0=none/a few, 1=most/all.

Religiosity. Adolescent-reported religiosity was the average of three items: religious beliefs are important, beliefs influence decisions, and is important that friends share beliefs, coded 1=strongly disagree, 2=disagree, 3=agree, 4=strongly agree (α=0.84).

Physical Health. For the parent and adolescent, ten diagnosed lifetime health conditions were summed: heart problems, diabetes, bronchitis or COPD, cirrhosis of the liver, hepatitis B or C, kidney disease, asthma, HIV or AIDS, cancer, high blood pressure (0=none, 1=1 condition, 2=2 or more conditions). Parents' and adolescents' self-reported general health was coded as 0=excellent, very good or good, 1=fair or poor.

Sociodemographics. Age (12-17 years for adolescents, 19-86 years for parents); gender (male, female); race/ethnicity (non-Hispanic white, non-Hispanic African American, Hispanic, Other); parental education (high school graduate or less, some college, college graduate); marital status (married, separated/divorced, widowed, never married); population density, as per residence in a Core Based Statistical Area (CBSA), defined by the Office of Management and Budget (not in CBSA, CBSA<1 million persons, ≥1 million persons). A CBSA is a US geographic area that consists of one or more counties (or equivalents) anchored by an urban center of at least 10,000 people plus adjacent counties that are socioeconomically tied to the urban center by commuting.

Adolescent race/ethnicity was used in the analyses of adolescent prescription opioid use.

Survey year. 2015, 2016, 2017.

The NSDUH implements extensive logical editing and statistical imputation procedures.[2] As a result, the substance use and sociodemographic variables have no missing data, and the physical health and psychosocial variables have very little missing data (range 0.3%-2.7%). In the multivariable model estimation, a category for missing was included, when necessary.

eTable 1. Prescription Opioid Pain Relievers Asked About by Class in National Survey on Drug Use and Health, 2015-2017

**Hydrocodone Products**
Vicodin®
Lortab®
Norco®
Zohydro® ER
Hydrocodone (generic)
**Oxycodone Products**
OxyContin®
Percocet®
Percodan®
Roxicet® (removed after 2015)
Roxicodone®
Oxycodone (generic)
**Tramadol Products**
Ultram®
Ultram® ER
Ultracet®
Tramadol (generic)
Extended-Release Tramadol (generic)
**Codeine Products**
Tylenol® with Codeine 3 or 4
Codeine Pills (generic)
**Morphine Products**
Avinza®
Kadian®
MS Contin®
Morphine (generic)
Extended-Release Morphine (generic)
**Fentanyl Products**
Actiq® (removed after 2015)
Duragesic®
Fentora®
Fentanyl (generic)
**Buprenorphine Products**
Suboxone®
Buprenorphine (generic)
Buprenorphine Plus Naloxone (generic) (added after 2015)
**Oxymorphone Products**
Opana®
Opana® ER
Oxymorphone (generic)
Extended-Release Oxymorphone (generic)
**Demerol®**
**Hydromorphone Products**
Dilaudid® or Hydromorphone (generic)
Exalgo® or Extended-Release Hydromorphone (generic)
**Methadone** (generic)
**Other**

©2021 Griesler PC et al. *JAMA Network Open*.

eTable 2. Prevalence of Past 12-Month Prescription Opioid (PO) Misuse With or Without a Prescription Among Selected Adult (n=1549) and Adolescent (n=1412) Misusers Based on Two Questions[a]

	**Misuse With-Prescription Only**	**Misuse With- and Without-Prescription**	**Misuse Without-Prescription Only**	**Missing**
**Respondent**	**% (95% CI)**	**% (95% CI)**	**% (95% CI)**	**% 95% CI**
**Adults**	30.3 (27.2-33.4)	29.4 (26.4-32.6)	36.6 (33.4-39.9)	3.8 (2.5-5.7)
**Adolescents**	16.2 (14.2-18.5)	32.4 (29.2-35.8)	40.6 (36.7-44.6)	10.8 (8.5-13.6)

[a]Includes adults aged 30 and over with a child under aged 18 living in the household and adolescents aged 12-17 living with a mother or father.
Two NSDUH questions:
Q1 - *PRYWAYS.* Which of these statements describe your use of pain relievers at any time in the past 12 months?
1. I used without a prescription of my own
2. I used in greater amounts than prescribed
3. I used more often than prescribed
4. I used for longer than prescribed
5. I used in some other way a doctor did not direct me to use

Q2 - *PRYGOT*. Now think again about the last time you used pain relievers in any way a doctor did not direct you to use. How did you use the pain reliever?
1. I got a prescription from just one doctor
2. I got prescriptions from more than one doctor
3. I stole from a doctor’s office, clinic, hospital, or pharmacy
4. I got from a friend or relative for free
5. I bought from a friend or relative
6. I took from a friend or relative without asking
7. I bought from a drug dealer or other stranger
8. I got in some other way

Three groups were defined: (1) misused with prescription only (Q1=2-5 and Q2=1-2); (2) misused with and without a prescription (Q1=1 and Q2=1-2 or Q1=2-5 and Q2=3-8); (3) misused without a prescription only (Q1=1 and Q2=3-8).

©2021 Griesler PC et al. *JAMA Network Open*.

eReferences

1. Center for Behavioral Health Statistics and Quality (CBHSQ), 2017 National Survey on Drug Use and Health Methodological Resource Book, Section 12: Questionnaire Dwelling Unit-Level and Person Pair-Level Sampling Weight Calibration, Substance Abuse and Mental Health Services Administration (SAMHSA). 2019, CBHSQ: Rockville, MD. https://www.samhsa.gov/data/sites/default/files/cbhsq-reports/NSDUHmrbQDUPairWgt2017/NSDUHmrbQDUPairWgt2017.pdf

2. Center for Behavioral Health Statistics and Quality (CBHSQ), 2017 National Survey on Drug Use and Health Methodological Resource Book, Section 10: Editing and Imputation Report, Substance Abuse and Mental Health Services Administration (SAMHSA). 2019: Rockville, MD. https://www.samhsa.gov/data/sites/default/files/cbhsq-reports/NSDUHmrbEditImputation2017.pdf

©2021 Griesler PC et al. *JAMA Network Open*.
